# [Corrigendum] The role of IGFBP-5 in mediating the anti-proliferation effect of tetrandrine in human colon cancer cells

**DOI:** 10.3892/ijo.2024.5681

**Published:** 2024-08-14

**Authors:** Ke Wu, Mi Zhou, Qiu-Xiang Wu, Shuang-Xu Yuan, Dong-Xu Wang, Jie-Li Jin, Jun Huang, Jun-Qin Yang, Wen-Juan Sun, Li-Hua Wan, Bai-Cheng He

Int J Oncol 46: 1205-1213, 2015; DOI: 10.3892/ijo.2014.2800

Following the publication of the above article, a concerned reader drew to the authors' attention that, among Figs. 1D, 2A and 4B, certain of the control western blots had been re-used in different blots.

The authors have retrieved and re-examined their original data, and were able to identify the correct control western blots where the data had been inadvertently duplicated in the affected original figures. The revised versions of Figs. 2 and 4, now featuring the correct control western blots, are shown in the subsequent two pages. The authors regret that the data in question featured in the original article had been re-used, and thank the Editor of *International Journal of Oncology* for granting them the opportunity to publish this corrigendum. All the authors agree with the publication of this corrigendum; furthermore, they apologize to the readership of the journal for any inconvenience caused.

## Figures and Tables

**Figure 2:**
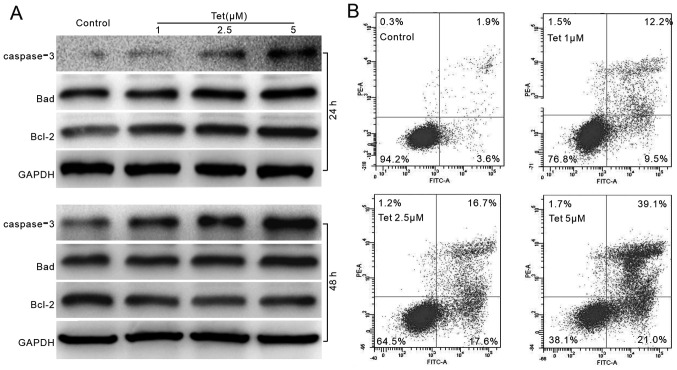
The effect of Tet on apoptosis in LoVo cells. (A) Western blot results show the effect of Tet on caspase-3, Bad and Bcl-2, GA PDH was used as loading control. (B) Flow cytometric analysis show the apoptosis induced by Tet. Each assay condition was done in triplicate.

**Figure 4:**
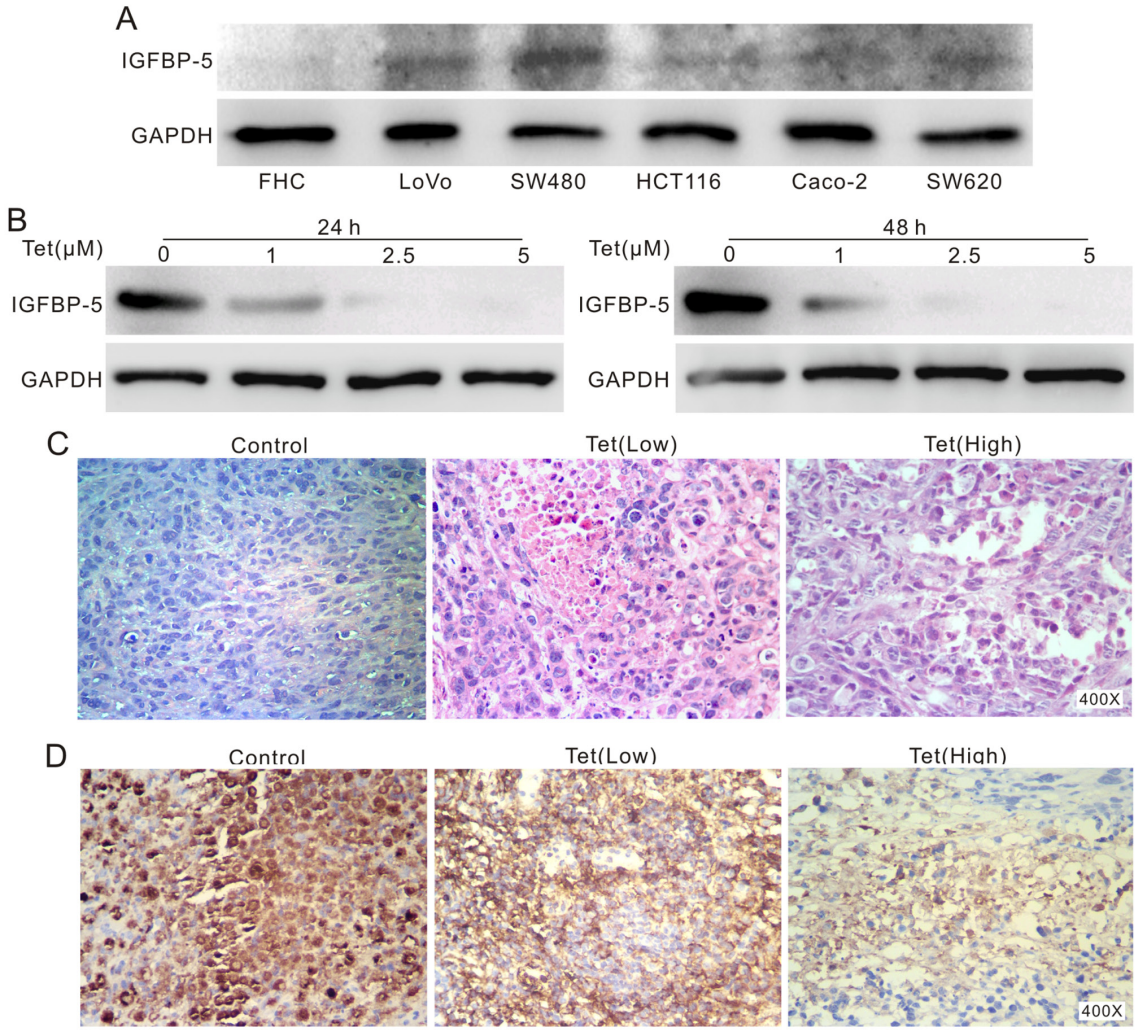
The effect of Tet on the expression of IGF BP-5 in colon cancer and tumor growth in xenograft colon cancer model. (A) Western blot assay shows the endogenous expression of IGF BP-5 in different colon cancer cells and FHC cells, GA PDH was used as loading control. (B) Western blot analysis shows the effect of Tet on the expression of IGF BP-5 in LoVo cells, GA PDH was used as loading control. (C) H&E staining shows Tet inhibits the colon cancer cell growth in the xenograft tumor model. (D) Immunohistochemical staining shows the effect of Tet on the expression of IGF BP-5 in a xenograft of colon cancer.

